# Auditory and Visual Statistical Learning Are Not Related to ADHD Symptomatology: Evidence From a Research Domain Criteria (RDoC) Approach

**DOI:** 10.3389/fpsyg.2018.02502

**Published:** 2018-12-21

**Authors:** Kaitlyn M. A. Parks, Ryan A. Stevenson

**Affiliations:** ^1^Department of Psychology, Western University, London, ON, Canada; ^2^Brain and Mind Institute, Western University, London, ON, Canada; ^3^Program in Neuroscience, Western University, London, ON, Canada; ^4^Department of Psychiatry, Western University, London, ON, Canada; ^5^Centre for Vision Research, York University, Toronto, ON, Canada

**Keywords:** inattention, hyperactivity, statistical learning, auditory, visual

## Abstract

Statistical learning is an implicit process that allows individuals to track and predict incoming events from their environment. Given that information is highly structured over time, events become predictable, allowing these individuals to make better sense of their environment. Among the studies that have examined statistical learning in attention deficit/hyperactivity disorder (ADHD), findings have been mixed. Our goal was to examine whether increased ADHD symptomatology related to decreased auditory and visual statistical learning abilities. To investigate this, we examined the entire range of ADHD symptomatology using a Research Domain Criteria approach with a clinically reliable questionnaire in addition to well-established auditory and visual statistical learning paradigms. Total ADHD symptomatology was not related to auditory and visual statistical learning. An identical pattern emerged when inattention and hyperactivity components were separated, indicating that neither of these distinct behavioral symptoms of ADHD are related to statistical learning abilities. Findings from the current study converge with other studies but go beyond finding a lack of a significant relationship – through Bayesian analyses, these data provide novel evidence directly supporting the hypothesis that ADHD symptomatology and statistical learning are decoupled. This finding held for overall levels of ADHD symptomatology as well as the subdomains of inattention and hyperactivity, suggesting that the ability to pick up on patterns in both auditory and visual domains is intact in ADHD. Future work should consider investigating statistical learning in ADHD across ages and beyond auditory and visual domains.

## Introduction

Similar events and pieces of information tend to co-occur reliably within the environment. The ability to identify and predict these statistical relationships in the environment is a process referred to as statistical learning. Extracting such probabilistic information is important for individuals to make sense of their world and has been shown to play a role in both language proficiency (Kidd, 2012) and predicting behavioral patterns (Baldwin et al., 2008). Statistical learning is an implicit mechanism that requires learners to process rapid and continuous transitions in sensory input. Individuals are able to learn patterns and regularities without explicit instruction or awareness (Reber, 1967). The ability to track and predict statistical patterns has been observed across many types of tasks and stimuli, including visual stimuli (Kirkham et al., 2002), tactile stimuli (Conway and Christiansen, 2005), non-linguistic sounds (Gebhart et al., 2009), auditory syllables (Saffran et al., 1996, 1999), and within scenes and in response to body movements (Baldwin et al., 2008).

Statistical learning has been explored in both children and adults with attention-deficit/hyperactivity disorder (ADHD) to test the hypothesis that impairments in statistical learning may contribute to ADHD symptomatology. Many of these studies have used an implicit sequence learning task known as the serial reaction time task (SRT). During this task, participants are required to respond to a set of stimuli that follows a repeating pattern that they should learn to predict with repeated exposure, assuming their statistical learning skills are intact. However, among the studies using the SRT with individuals with ADHD, findings have been inconsistent, with some studies reporting intact (Karatekin et al., 2009; Vloet et al., 2010; Takács et al., 2017; Pedersen and Ohrmann, 2018), and others reporting atypical learning relative to typically developing peers (Klorman et al., 2002; Barnes et al., 2010). Despite these findings, there is evidence to suggest that the ability to learn implicit patterns is impaired outside of traditional SRT tasks, suggesting that SRT tasks may fall short in capturing impairments. For instance, impaired statistical learning has been reported in preschoolers with ADHD when an artificial grammar task was used (Domuta and Pentek, 2003) and individuals with ADHD also tend to have difficulties arranging or sequencing information in a way that is logical and cohesive (Purvis and Tannock, 1997). One possible limitation to using the SRT task to measure implicit sequence learning is that only response time is used as an index of learning (Moisello et al., 2009), making it difficult to disentangle implicit and explicit processes. To test whether statistical learning abilities outside of the SRT task relate to ADHD symptomatology, we used well-established auditory and visual statistical learning paradigms which have the benefit of reliably assessing implicit learning through the collection of accuracy scores.

Given the apparent difficulties with statistical learning in ADHD, we hypothesize that:

(1) ADHD symptomatology will be inversely related to auditory and visual statistical learning abilities and,
(2) Given that inattention and hyperactivity are characterized as distinct behavioral symptoms in ADHD, we predict that inattention and hyperactivity will uniquely contribute to statistical learning abilities.

## Materials and Methods

Participants included 104 young adults recruited from the undergraduate psychology pool at the University of Western Ontario as part of a larger study on developmental disabilities. Six participants were excluded, five for being over the maximum age for which the standardized language measure intended, and one for failure to complete the protocol, resulting in a final sample of 98 participants aged 16–21 years (*Mean age* = 18.19, *SD* = 0.74 years, 64.3% female). Participants were recruited based upon the Research Domain Criteria (RDoC) framework in order to cover the entire range of symptomatology (Insel et al., 2010; Cuthbert and Insel, 2013), and thus were not required to have a formal ADHD diagnosis. All participants were English speaking and had self-reported normal or corrected-to-normal vision and normal hearing. All protocols were approved by the University of Western Ontario Research Ethics Board.

### Auditory Statistical Learning Paradigm

Participants completed a well-established auditory statistical learning task similar to that described in the study by Saffran et al. (1997). Participants first completed a language exposure phase where they were exposed to a structured, unsegmented language stream for 21 min. The language consisted of six tri-syllabic nonsense “words”: *tutibu, babupu, bupada, pidadi, patubi, and dutaba.* There were no acoustic markers to indicate word boundaries between words. However, within the language stream, there were higher transitional probabilities within words (1.0 or 0.33) than between words (0.1 or 0.2), where transitional probabilities were calculated as:

$$p(Y|X)=\frac{p\left(X\cap Y\right)}{p\left(X\right)}$$

Immediately following the artificial language exposure, participants completed a two-alternative forced-choice test (2AFC) to assess whether they could identify trained words from the artificial language (see Supplementary Material for auditory and visual statistical learning trial details).

### Visual Statistical Learning Paradigm

**FIGURE 1:**
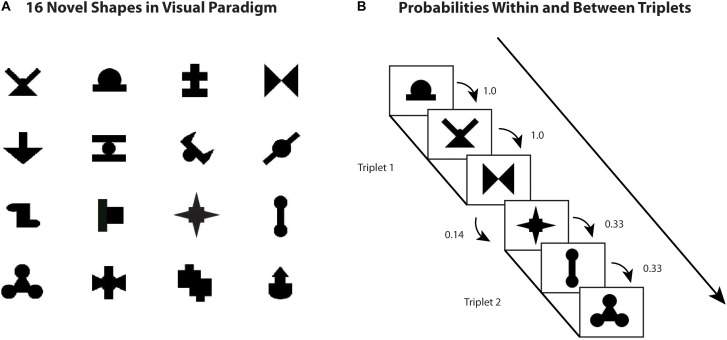
Visual statistical learning task figures **(A)** 16 novel shapes used in visual paradigm **(B)** example of probabilities within and between triplets.

Participants then completed a visual statistical learning task identical to that used by Siegelman et al. (2016). Similar to the auditory task, the visual task was comprised of both a learning phase and a test phase where transitional probabilities were higher for within-shape sequences than between (Figure 1). The shapes were organized into eight triplets that differed according to their within-triplet transitional probabilities, with four triplet items having transitional probabilities of 0.33 and the other four having transitional probabilities of 1.0. Between-triplet transitional probabilities were 0.14 or less. In the 42-item test phase, the first block of 34 four trials were pattern recognition items and required participants to pick the familiar sequence from either two (2AFC) or four (4AFC) possible responses. The next eight trials were pattern completion items where participants were instructed to select the shape that best completed the sequence.

### Standardized Measure of ADHD Symptomatology

The Adult ADHD Self-Report Scale (ASRS-v1.1; Kessler et al., 2005) was used to measure participants’ ADHD symptoms. On this 18-item measure, participants responded on a five-point Likert scale to reflect the frequency with which they experienced challenges with inattention and/or hyperactivity. The ASRS was chosen for the current study because it is a reliable screening measure that has been shown to be successful in screening for ADHD characteristics in the general population where no formal diagnosis of ADHD has been reported (Kessler et al., 2005, 2007).

### Analysis

Participants’ mean accuracy scores were calculated for both statistical learning paradigms and *t*-tests were conducted to compare accuracy scores to chance levels to ensure that statistical learning occurred. ADHD symptomatology was quantified by scoring participants’ responses for each of the 18-items of the ASRS as “0” or “1” in accordance with Kessler et al. (2005) to derive a total score (sum of scores across all 18 items), and scores for inattention (sum of items tapping inattention) and hyperactivity (sum of items tapping hyperactivity).

Bivariate Bayesian correlations (JASP Team, 2018) were then used to examine relationships between ADHD symptoms and statistical learning with a Benjamini-Hochberg false discovery rate procedure (*Q* = 0.1) used to correct for multiple comparisons. Bayesian linear regressions were also used to examine whether inattention and hyperactivity uniquely contributed to statistical learning.

## Results

Scores on the ASRS confirmed that the sample spanned nearly the entire range of symptomatology, (0–17 out of a possible 0–18) in line with the RDoC framework. Previous standardization of the ASRS identified scores of 0–10/11–18 as the best split point predicting clinical-level symptoms with a classification accuracy of 96.2% (specificity = 98.3%, sensitivity 56.3%) (Kessler et al., 2007). The current sample included 16 participants (16.33%) scoring at levels considered at risk for ADHD.

Above chance performance on statistical learning tasks were examined and found to be reliability above chance for both auditory (mean accuracy = 64%, *t*_(97)_ = 58.53, *p* < 0.001, *d* = 5.82) and visual (mean accuracy = 58%, *t*_(97)_ = 42.07, *p* < 0.001, *d* = 4.14) statistical learning tasks.

**FIGURE 2:**
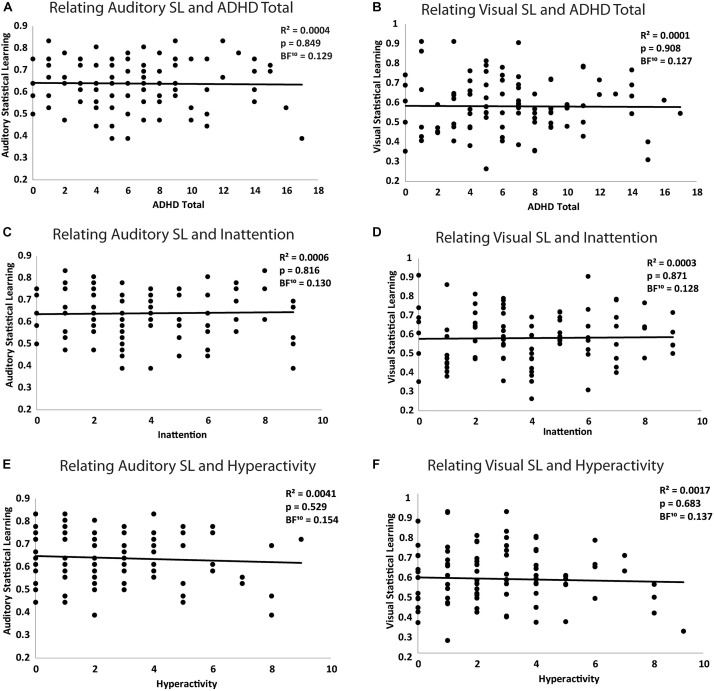
Relating **(A)** auditory statistical learning and total ADHD symptomatology **(B)** visual statistical learning and total ADHD symptomatology **(C)** auditory statistical learning and inattention **(D)** visual statistical learning and inattention **(E)** auditory statistical learning and hyperactivity **(F)** visual statistical learning and hyperactivity where total ADHD symptomatology, inattention, and hyperactivity were all assessed using the ASRS.

Contrary to our predictions, auditory and visual statistical learning were not significantly related to overall ADHD symptomatology (*r*_(97)_ = -0.02, *p* = 0.849, *BF*_10_ = 0.129, and *r*_(97)_ = -0.01 *p* = 0.908, *BF*_10_ = 0.127, respectively). Further, when isolating inattention versus hyperactivity characteristics of ADHD, neither inattention (*r*_(97)_ = 0.02, *p* = 0.816, *BF*_10_ = 0.130; *r*_(97)_ = 0.02, *p* = 0.871, *BF*_10_ = 0.128) nor hyperactivity (*r*_(97)_ = -0.06, *p* = 0.529, *BF*_10_ = 0.154, *r*_(97)_ = -0.04, *p* = 0.683, *BF*_10_ = 0.137) scores significantly related to auditory or visual statistical learning, respectively (Figure 2). To determine whether inattention and hyperactivity uniquely contributed to auditory statistical learning, a multiple regression was performed, and the overall model was not significant, *F*_(2,95)_ = 0.417, *p* = 0.660, *R*^2^ = 0.01, *BF*_10_ = 0.095. When isolated, neither inattention (β = 0.003, *t*_(95)_ = 0.66, *p* = 0.510, *sr*^2^ = 0.005, *BF*_10_ = 0.218) nor hyperactivity β = -0.005, *t*_(95)_ = -0.88, *p* = 0.379, *sr*^2^ = 0.008, *BF*_10_ = 0.254) were statistically significant in this model. A second multiple regression was performed to determine if inattention and hyperactivity uniquely contributed to visual statistical learning and the overall model was not significant, *F*_(2,95)_ = 0.181, *p* = 0.835, *R*^2^ = 0.004, *BF*_10_ = 0.078. When isolated, both inattention (β = 0.003, *t*_(95)_ = 0.44, *p* = 0.659, *sr*^2^ = 0.002, *BF*_10_ = 0.215) and hyperactivity (β = -0.004, *t*_(95)_ = -0.58, *p* = 0.564, *sr*^2^ = 0.003, *BF*_10_ = 0.229) were not statistically significant in this model.

## Discussion

Although our hypotheses surrounding ADHD symptomatology and statistical learning were not supported by the data, past research on this topic has been mixed, with a number of studies suggesting that individuals with ADHD exhibit impaired statistical learning abilities (Klorman et al., 2002; Domuta and Pentek, 2003; Barnes et al., 2010; Sakreida, 2011). Here, we hypothesized that individuals with higher ADHD symptomatology would show decreased statistical learning abilities, however, we did not find any evidence to support a relationship between ADHD symptoms and statistical learning.

It is notable that despite using a variety of tasks to assess statistical learning, a number of studies have found this ability to be unimpaired in ADHD, suggesting that similar results would emerge if a clinical sample was included in the current study. Despite this, there is evidence to suggest that statistical learning may be impaired in ADHD, including impairments in executive functioning (Willcutt et al., 2005) and abnormalities in the brain structures that underlie the implicit memory system, such as the frontal and basal-ganglia networks (Cubillo et al., 2012). This system is particularly important for processing sequences and predicting probabilistic outcomes within these sequences (Takács et al., 2017). Indeed, children with ADHD have been shown to be less sensitive at the neural level to violations in sequences that follow a probabilistic structure (Klorman et al., 2002). Finally, it has been hypothesized that the neural circuits responsible for predicting probabilistic cues in the environment including *what* (frontostriatal) may occur and *when* (frontoneocerebellar) that event may occur are both impaired in ADHD, resulting in less accurate expectations about the environment as well as a weakened ability to detect violations and adjust one’s behavior according to these violations (Nigg and Casey, 2005).

Prior studies failing to find a significant link between statistical learning and ADHD symptoms, however, have been limited in that common frequentist hypothesis testing is unable to provide direct support for a null hypothesis. Our Bayesian analyses provide novel evidence directly supporting the hypothesis that ADHD symptomatology and statistical learning are decoupled, at least in individuals who do not have a formal diagnosis of ADHD. This finding held for overall levels of ADHD symptomatology as well as the subdomains of inattention and hyperactivity. These null findings are important for informing future studies that intend to assess if/how this ability relates to ADHD and related symptoms.

There are several important considerations for future work. First, future studies exploring the possible relationship between ADHD symptomatology and statistical learning should include child participants to determine whether the relations differ in younger populations. Previous work examining statistical learning in children with ADHD has found this ability to be impaired (Domuta and Pentek, 2003). Further, the majority of research examining statistical learning abilities in typical development has focused on child populations (Newman et al., 2006; Kidd, 2012; Ellis et al., 2014; Kidd and Arciuli, 2016). It is therefore possible that the ability to learn these patterns is more prominent in the early years. Second, future research should examine whether the ability to learn more complex sequencing relationships that involve non-adjacent dependencies is impaired in ADHD. Finally, an RDoC approach should be applied to future work to examine the entire spectrum of levels of ADHD symptomatology. Online recruitment platforms, such as Amazon Mechanical Turk can help researchers achieve this goal by reaching a more representative population with a distribution of symptoms across a continuous spectrum of ADHD (Crump et al., 2013).

## Author Contributions

KP and RS both played a significant role in all aspects of this project including the experimental design, entry, analysis, interpretation of data, the drafting and revising of this manuscript, and final approval for publication.

## Conflict of Interest Statement

The authors declare that the research was conducted in the absence of any commercial or financial relationships that could be construed as a potential conflict of interest.
