# Supplementary material for: Auditory and Visual Statistical Learning Are Not Related to ADHD Symptomatology: Evidence From a Research Domain Criteria (RDoC) Approach
Source: Front Psychol. 2018 Dec 21;9:2502. doi: 10.3389/fpsyg.2018.02502 (PMC6308122; doi:10.3389/fpsyg.2018.02502)
Supplement: Supplementary file 1 [file Table_1.docx]

Supplementary Material

Auditory and Visual Statistical Learning are not Related to ADHD Symptomatology: Evidence from a Research Domain Criteria (RDoC) Approach

Kaitlyn M.A Parks Ryan A. Stevenson

*** Correspondence:** Kaitlyn M.A Parks: kparks7@uwo.ca

**Auditory statistical learning paradigm**

The language exposure phase consisted of 360 tokens of each word in random order, with no word presented twice in sequence. The language maintained a consistent speech rate (average 5.1 syllables/s) and was normalized to a pitch of F0 = 196 Hz. For each of the 36 test items, participants heard a trained word from the artificial language paired with a nonword foil, separated by 500ms of silence. Nonword foils were constructed from the same syllable inventory as the words from the artificial language, where transitional probabilities between syllables were 0.0: *pubati, tapudi, dupitu, tipabu, bidata, batipi*. For the test phase, instructions were displayed on the computer screen, asking participants *‘which of these two words sounds most like something you heard in the language’* where they then indicated their response on a keyboard by pressing the “*A*” or “*L*” key for the first or second “word”, respectively. Overall accuracy on the 2AFC test phase were analyzed.

**Visual statistical learning paradigm**

During the learning phase, participants passively viewed the stream of shapes for 10 minutes, with each triplet sequence appearing 24 times. Shapes were presented for 800ms, with a 200ms inter-stimulus interval (ISI). The test phase began immediately and consisted of 42 test items of varying difficultly. The first block of 34 four trials were pattern recognition items. Pattern recognition items included both pairs of shapes (part triplets) and triplets. Participants were instructed to *‘choose the pattern that appeared together in the first part’* for 24 triplet items, sixteen being 2AFC and eight 4AFC and *‘choose the pattern that you are most familiar with as a whole’* for ten part-triplet items, six being 2AFC and four 4AFC. All responses were numbered, and participants indicated their response by clicking the corresponding key on a keyboard. The next eight trials were pattern completion items. For these trials, participants were shown an incomplete trained sequence, and selected the shape that best completed the sequence from a set of three possible responses. Participants were instructed to *‘choose the shape that best completes the pattern’* from the 3AFC items, which included four triplet-completion items and four pair-completion (part-triplet) items. For all test trials, there was only one triplet or part-triplet that followed the statistical regularities presented in the test phase. Accuracy scores were analyzed.
